# Detection and Comparative Analysis of Methylomic Biomarkers of Rheumatoid Arthritis

**DOI:** 10.3389/fgene.2020.00238

**Published:** 2020-03-27

**Authors:** Xin Feng, Xubing Hao, Ruoyao Shi, Zhiqiang Xia, Lan Huang, Qiong Yu, Fengfeng Zhou

**Affiliations:** ^1^Department of Epidemiology and Biostatistics, School of Public Health, Jilin University, Changchun, China; ^2^Jilin Institute of Chemical Technology, Jilin, China; ^3^BioKnow Health Informatics Lab, College of Computer Science and Technology, and Key Laboratory of Symbolic Computation and Knowledge Engineering of Ministry of Education, Jilin University, Changchun, China; ^4^BioKnow Health Informatics Lab, College of Software, and Key Laboratory of Symbolic Computation and Knowledge Engineering of Ministry of Education, Jilin University, Changchun, China; ^5^BioKnow Health Informatics Lab, College of Life Sciences, Jilin University, Changchun, China; ^6^College of Computer Science and Technology, and Key Laboratory of Symbolic Computation and Knowledge Engineering of Ministry of Education, Jilin University, Changchun, China

**Keywords:** feature selection, rheumatoid arthritis, methylation biomarker, methylome, chromosome Y

## Abstract

Rheumatoid arthritis (RA) is a common autoimmune disorder influenced by both genetic and environmental factors. To investigate possible contributions of DNA methylation to the etiology of RA with minimum confounding genetic heterogeneity, we investigated genome-wide DNA methylation in disease-discordant monozygotic twin pairs. This study hypothesized that methylomic biomarkers might facilitate accurate RA detection. A comprehensive series of biomarker detection algorithms were utilized to find the best methylomic biomarkers for detecting RA patients using the methylomic data of the peripheral blood samples. The best model achieved 100.00% in accuracy (Acc) with 81 methylomic biomarkers and a 10-fold cross-validation (10FCV) strategy. Some of the methylomic biomarkers were experimentally confirmed to be associated with the onset or development of RA. It is also interesting to observe that many of the detected biomarkers were from chromosome Y, supporting the knowledge that RA has a significant gender discrepancy.

## Introduction

The chronic autoimmune disease rheumatoid arthritis (RA) demonstrates significant changes to joints, with major symptoms like joint pain and swollenness (Triantafyllias et al., 2016). RA is strongly associated with the inflammation around major organs like lungs (Chatzidionisyou and Catrina, 2016; Farquhar et al., 2019) and heart (Crowson et al., 2013; Lazzerini et al., 2017). RA may be developed in about 1% of the population in the developed countries (Smolen et al., 2016). Moreover, females have a 2.5 times high risk than males to develop RA (Alam et al., 2011).

The cause of RA remained unclear and was hypothesized to be under the orchestrated regulation of both genetic and epigenetic factors (Villanueva-Romero et al., 2018; Khan et al., 2019). Various genetic biomarkers were detected through genome-wide association studies (Massey et al., 2018; Shadrina et al., 2018; Lopez-Mejias et al., 2019). Multiple genetic mutations were detected to be statistically associated with the susceptibility for RA, including the SNPs in the genes interferon regulatory factor 4 (IRF-4) (Lopez-Isac et al., 2016) and Solute Carrier family 8 (SLC8A3) (Julia et al., 2016). Genetic factors were also observed to be associated with the treatment responses of the tumor necrosis factor alpha inhibitors (TNFi) (Massey et al., 2018) and the methotrexate (MTX) monotherapy (Taylor et al., 2018).

Recent studies also demonstrated that the differential status of the epigenomic loci was also statistically significantly associated with RA even in a small population (Julia et al., 2017; Carnero-Montoro and Alarcon-Riquelme, 2018). The RA pathogenesis was observed to be actively regulated by the epigenetic modifications of the immune machineries in the joint tissues (Ibanez-Cabellos et al., 2019). Various environmental factors like cigarette smoking and certain oral pathogens may induce RA through epigenetic modifications (Brandt et al., 2019). Novel treatment plans were proposed to use epigenetic modulators to reverse the differentially methylated regions (Petralia et al., 2019). So the detection of RA methylation biomarkers may both facilitate the understanding of RA pathogenesis and propose more epigenetic drug targets.

There were two main types of computer algorithms to detect biomarkers, i.e., filters and wrappers (Xie et al., 2013; Singh et al., 2018; Verde and De Pietro, 2019). A filter tries to rank the features by each feature’s statistical association significance with the phenotype, assuming the features are independent of each other (Lyu et al., 2017). The filter algorithm has a linear time complexity and runs fast enough for many large datasets (Xu et al., 2018). A wrapper utilizes a few heuristic rules to generate a feature subset with a performance evaluation iteratively, and the final feature subset is output if the stop criterion is met (Tekin Erguzel et al., 2015). The strategies of both filters and wrappers may be integrated to generate a hybrid feature selection algorithm (Kumar and Nirmalkumar, 2019; Wu et al., 2019).

This study hypothesized that methylomic features might reflect both the genetic and epigenetic status of RA. So a comprehensive biomarker detection procedure was carried out to find a biomarker set with the satisfying RA prediction accuracy (Acc). The best RA prediction model was also compared with the two sets of methylomic biomarkers from the previous studies. Our model demonstrated a better RA prediction Acc and interesting biological observations.

## Materials and Methods

### Summary of the Dataset

This study screened 485,577 methylomic features detected from 79 RA children and their 79 healthy monozygotic twin siblings (Webster et al., 2018). The twin pairs were identified from the TwinsUK register (Moayyeri et al., 2013) and the RA status was detected in a questionnaire between 1997 and 2002. The twin volunteers were recruited after an advertisement in the National RA Society newsletter in 2013. The RA status was clinical confirmed after these twins were recruited, and only those twins with one healthy and the other RA status were kept for this study. The blood samples were stored at −80°C for DNA extraction.

The methylome was generated by the Illumina HumanMethylation450 BeadChip 15017482 v1.1. The raw data were available at the ArrayExpress database (Athar et al., 2019) with the accession number E-MTAB-6988. This methylomic dataset was formulated as a binary classification problem between the pediatric RA patients and the controls.

The data were provided in the raw format of IDAT, and the methylation level was calculated using the function getBeta() of the R package minfi version 1.28.3 (Aryee et al., 2014).

### Pre-screening the Methylomic Features

Many feature selection algorithms run slow on a large dataset, and each methylome has almost half a million features. The downstream feature selection algorithms may crash if they were used directly on the methylomic datasets. So we carried out a pre-screening step to reduce the number of features to be within the capacity of the feature selection algorithms. So the classifier LinearSVC was used to select features for further feature screening. The Python package sklearn has a module SelectFromModel() for this purpose. The model can select features based on the indicators given by the LinearSVC trained on the dataset and the user may determine the number of features screened for further analysis.

### Filter Algorithms

Four widely used filter algorithms were used to rank the features, assuming the features were independent of each other. *T*-test (Ttest) assumed that the data followed a normal distribution and were widely used in bioOMIC data. Ttest evaluated the statistical significance of a feature’s differential values between two groups of samples (Kim, 2015; Gharbali et al., 2018; Jankowski et al., 2018). This study focused on the differential methylated residues between the RA patients and the siblings and assumed the independences between the two groups of samples (Lotsch et al., 2013; Kahl et al., 2018).

Chi-squared test (Chi2) can be used to select features with the highest values of the chi-squared statistics from a vector × relative to the classes. The chi-square test measures dependence between stochastic variables. It also checked whether a feature was statistically significantly associated with the class label under the assumption of a chi-squared distribution (Bangdiwala, 2016; Fernandez Rojas et al., 2019).

Mutual information (MI) measured the mutual dependency between a feature and the class label (Wei and Stocker, 2016; Meng et al., 2019). MI is equal to zero if and only if two random variables are independent, and a higher value means a higher dependency between the two random variables. The function relies on non-parametric methods based on entropy estimation from k-nearest-neighbor (KNN) distances.

Pearson correlation coefficient (PCC) evaluated the linear correlation between a feature and the class label with the assumption of sample independence (Liu et al., 2017). The PCC measures the linear relationship between two variables. PCC assumed that each variable be normally distributed, and do not necessarily have a zero-mean. Like the other correlation coefficients, PCC varies between −1 and +1 with 0 implying no correlation between the two variables. Correlations of −1 or +1 imply an exact negative or positive linear relationship. Positive correlations imply that as *x* increases, so does *y*. Negative correlations imply that as *x* increases, *y* decreases. The *p*-value roughly indicates the probability of an uncorrelated system producing variables that have a Pearson correlation at least as extreme as the one computed from these variables.

### Recursive Feature Elimination Strategy

Recursive feature elimination (RFE) was a strategy to iteratively remove a feature with the least weight from the training of a classification model. The following four classification models were used to build the RFE feature selection procedure. Logistic regression (LR) (rfeLR) was a popular binary classifier and may be embedded in the RFE strategy (Pandey et al., 2018). LR is also known in the literature as logit regression, maximum-entropy classification (MaxEnt), or the log-linear classifier. In this model, the probabilities describing the possible outcomes of a single trial are modeled using a logistic function.

Lasso was a regression model and may be used to assign weights to features after a model training (rfeLasso) (Wang et al., 2019). The Lasso is a linear model that estimates sparse coefficients. It is useful in some contexts due to its tendency to prefer solutions with fewer non-zero coefficients, so Lasso can effectively reduce the number of features upon which the given solution is dependent. For this reason, Lasso and its variants are fundamental to the field of compressed sensing (Angelosante et al., 2009). Mathematically, it consists of a linear model with an added regularization term. The objective function to minimize is:

$$\min_{w}\frac{1}{2\,n_{\text{samples}}}\,{||X_{w-y}||}_{2}^{2}+\alpha \,{||w||}_{1}.$$

The lasso estimate thus solves the minimization of the least-squares penalty with α*w*_1_ added, where α is a constant and *w*_*1*_ is the l1-norm of the coefficient vector.

The Naïve Bayes method calculated the association probability of each feature with the class label under the assumption of inter-feature independence (rfeNBayes) (Youn and Jeong, 2009). Naive Bayes methods are a set of supervised learning algorithms based on applying Bayes’ theorem with the “naive” assumption of conditional independence between every pair of features given the value of the class variable. Naive Bayes learners and classifiers can be extremely fast compared to more sophisticated methods. The decoupling of the class conditional feature distributions means that each distribution can be independently estimated as a one-dimensional distribution. This in turn helps to alleviate problems stemming from the curse of dimensionality.

The ridge regressor (rfeRidge) tried to assign minimized weights to non-associated features to a model (Barker and Brown, 2001; Rottmann and Berbeco, 2014). Ridge regression addresses some of the problems of ordinary least squares by imposing a penalty on the size of the coefficients. The ridge coefficients minimize a penalized residual sum of squares:

$$\min_{w}{||X_{w-y}||}_{2}^{2}+\alpha \,{||w||}_{2}^{2}.$$

The complexity parameter α ≥ 0 controls the amount of shrinkage: the larger the value of α, the greater the amount of shrinkage and thus the coefficients become more robust to collinearity.

### Heuristic Feature Selection Strategies

Three heuristic feature selection strategies were used to generate a feature subset. The ascending feature screening (AFS) strategy started with an empty feature subset and selected the next feature with the best rank or largest weight after a model training. Then this chosen feature was removed from the remaining feature list. While the descending feature screening (DFS) strategy started with all the features and removed the next feature with the lowest rank or the least weight after a model training. Cawley and Talbot (2010) suggested that a classification model may be over-fitted if the number of training samples was smaller than that of features. We proposed a feature removal procedure BackFS to carry out an iterative removal of a feature that contributed the least prediction performance improvement. The feature subset with the best prediction performance was kept for further analysis.

All the computational experiments were conducted in the Python programming language version 3.6.5. Chi2 and MI were provided in the python sklearn version 0.19.1. PCC and Ttest were provided in the python scipy version 1.1.0. The four RFE procedures were programmed using the python sklearn version 0.19.1.

### Classification Algorithms

Five widely used classifiers were utilized to measure the prediction performance of a feature subset. The discriminative power of a feature subset may be evaluated by a multivariate LR (Inzaule et al., 2018). The support vector machine (SVM) with the linear kernel function was another binary classifier that had been widely used for biomedical datasets (Citak-Er et al., 2018). SVMs are a set of supervised learning methods used for classification, regression, and outlier detection which can analyze data in classification and regression analysis. Given a set of training instances, each training instance is marked as belonging to one of the two categories, and the SVM training algorithm creates a model that assigns new instances to one of the two categories, making it a non-probability two Meta linear classifier. The SVM model represents instances as points in space, so that the mapping allows the instances of the individual categories to be separated by as wide an apparent interval as possible. Then, map new instances to the same space and predict which category they belong to based on which side of the interval they fall on. SVM may also be used to select biomarkers. After an SVM model was trained on a dataset, each input feature was assigned with a weight and the features with the default weight threshold 1*e*−5 may be chosen for further analysis.

The simple classifier KNN had demonstrated very good prediction accuracies in some cases (Nejadgholi and Bolic, 2015; Yang et al., 2017). Neighbors-based classification is a type of instance-based learning or non-generalizing learning. It does not attempt to construct a general internal model, but simply stores instances of the training data. Classification is computed from a simple majority vote of the nearest neighbors of each point: a query point is assigned the data class which has the most representatives within the nearest neighbors of the point.

The ensembled classifier Random Forest (RF) integrated the final decision based on the prediction results of multiple random trees (Lu et al., 2017; Olsen et al., 2018; Rahman et al., 2018). The RandomForest algorithm is perturb-and-combine techniques specifically designed for trees. This means a diverse set of classifiers is created by introducing randomness in the classifier construction. The prediction of the ensemble is given as the averaged prediction of the individual classifiers. In RFs, each tree in the ensemble is built from a sample drawn with replacement (i.e., a bootstrap sample) from the training set. The Gaussian naïve Bayes classifier was used in this study as an evaluator of a feature subset (Cao et al., 2017). GaussianNB implements the Gaussian Naive Bayes algorithm for classification. The likelihood of the features is assumed to be Gaussian:

$$P\,\left(x_{i}|y\right)=\frac{1}{\sqrt{2\,\pi \,\sigma_{y}^{2}}}\,\text{exp}\,\left(-\frac{{\left(x_{i}-\mu_{y}\right)}^{2}}{2\,\sigma_{y}^{2}}\right).$$

The parameters σ*_*y*_* and μ*_*y*_* are estimated using maximum likelihood.

The python sklearn version 0.19.1 provided the code of these five classifiers.

### Performance Measurements

Three classification performance measurements, i.e., accuracy (Acc), sensitivity (Sn), and specificity (Sp), were used to evaluate how well a feature subset performed (Ye et al., 2017; Xu et al., 2018; Yokoi et al., 2018; Zhao et al., 2018). The RA children were regarded as the positive samples (P) while the matched controls were the negative samples (N). P and N were also denoted as the numbers of positive and negative samples. Sensitivity (Sn) was defined as the correctly predicted ratio of positive samples, i.e., Sn = TP/(TP + FN) = TP/P, where TP and FN were the numbers of correctly and incorrectly predicted positive samples, respectively. Specificity (Sp) was the correct prediction ratio of negative samples, i.e., Sp = TN/(TN + FP) = TN/N, where TN and FP were the numbers of negative samples with correct and incorrect predictions, respectively. The overall prediction Acc was defined as Acc = (TP + TN)/(P + N).

These measurements were used in various prediction models like the DNA and RNA functional elements (He et al., 2018; Feng et al., 2019). And they were calculated using the 10-fold cross-validation (10FCV) strategy as similar in Ye et al. (2017) and Zhao et al. (2018).

### Experimental Design

**FIGURE 1:**
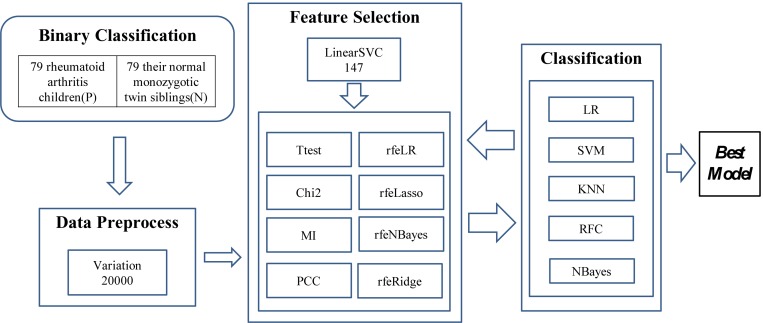
Experiment flowchart of this study. Three major steps were carried out to find the best classification model. The first step was to find the 20,000 features with the largest variation. Then a subset of 147 features was detected using LinearSVC, and 10 feature selection algorithms were utilized to find a better feature subset. The prediction performance was evaluated using five popular binary classifiers.

The experiments were carried out in three major steps, as illustrated in Figure 1. The first step was to find 20,000 features with the largest variations. A methylation residue with a large variation was easier to be detected while a residue with a stable methylation level required a high-resolution technology to measure. And the downstream feature selection algorithms may crash on a dataset with a large number of features. So we have to reduce the feature dimensions to be within the capacity of the eight feature selection algorithms. So LinearSVC was used to select 147 features for further feature screening.

Then the two steps of feature selection and classification were carried out iteratively to find the best classification model using the selected features, as shown in Figure 1.

## Results and Discussion

### Data Preprocessing

The raw data of this methylomic dataset was provided in the format IDAT, and was processed using the function getBeta() of the R package minfi version 1.28.3 (Aryee et al., 2014). There were 485,577 methylation features for each sample, among which 65 probes designed to interrogate SNPs within the samples and was ignored in the R package minfi. Some methylation residues had many missing values, e.g., the feature cg01550828 has no values in all the 158 samples. The feature cg01550828 was a cysteine in the N termini of the gene Ring Finger Protein 168 (RNF168), which encoded an E3 ubiquitin ligase protein. After the preprocessing, 485,511 methylomic features were detected for the following analysis.

We hypothesized that methylated residues with larger beta-value fluctuations may be easier to detect in the clinical practice. Therefore, we calculated the standard deviation of the beta-values of each methylated residue, and sorted the features in the descendental order. The top-ranked 20,000 features of the 158 samples were kept for further analysis.

### Limitations the Variation Threshold 20,000

**FIGURE 2:**
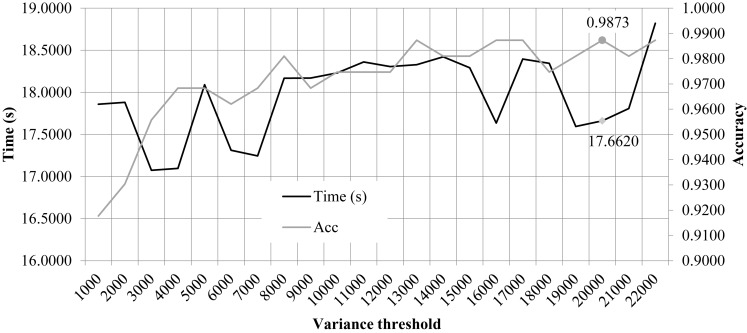
Classification accuracy and time cost of the classifier LinearSVC with different variance thresholds. The horizontal axis was the “variance threshold.” The left and right vertical axises were the computational time cost (seconds) and the classification accuracy, respectively.

We performed the 10FCV of the classifier LinearSVC on the features with different variation thresholds, as shown in Figure 2. Due to that the number of features were much larger than the number of samples, only the features with the LinearSVC model weight larger than the default weight threshold 1*e*−5 were kept for model performance evaluation. Figure 2 demonstrated the running time and 10FCV classification Acc of different numbers of features, i.e., 1000, 2000, 3000, …, 22,000. As shown in the figure, the variance threshold 20,000 achieved 0.9873 in Acc while costed a very relatively small running time 17.6620 s. But the procedure of feature selection and classification was not optimized for the final classification Acc. So the other choice of variance threshold may achieve a better final classification Acc.

The evaluation procedure was carried out in a computer with the Windows 7 operating system and Python 3.7 programming language. The computer had a 3.30GHz CPU, 32 Gb memory, and 1Tb hard disk.

### Optimizing LinearSVC to Select Features

**FIGURE 3:**
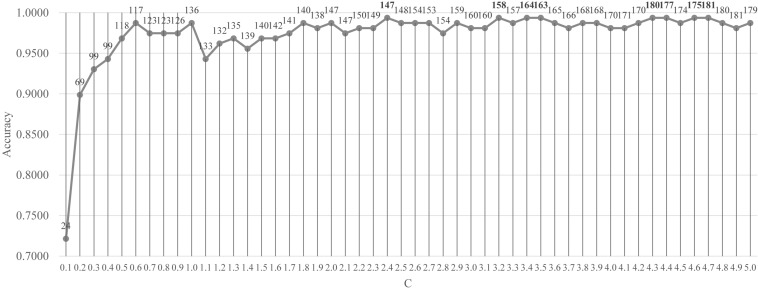
Classification performance of the classifier LinearSVC with different values of the penalty parameter *C*. The parameter *C* was screened by the values between [0.10, 5.00] with the step size 0.10. The vertical axis was for the classification accuracy and the horizontal axis was for the values of the parameter *C*. The number of features selected was given on top of each point in the curve.

Firstly, the feature selection procedure SelectFromModel() was used to find the initial feature subset with a reasonable prediction accuracy, as shown in Figure 3. The screening procedure was provided by the Python package scikit-learn version 0.21.2 and Python version 3.6. The penalization was carried out by the L1 penalty. In the Python package sklearn.svm.LinearSVC, the parameter *C* was a float with default = 1.0. It was a regularization parameter. The strength of the regularization was inversely proportional to *C* and this parameter must be strictly positive. The parameter *C* was screened by the values between [0.10, 5.00] with the step size 0.10, as shown in Figure 3.

Figure 3 demonstrated that after *C* reached the value 1.8, the prediction accuracy remained stable. The classifier LinearSVC achieved Acc = 0.9873 with *C* = 1.8 and 140 features. The best prediction accuracy 0.9937 was achieved by *C* = 2.4, 3.2, 3.4, 3.5, 4.3, 4.4, 4.6, and 4.7. The data demonstrated that the best Acc = 0.9937 was achieved by many choices of the parameter *C*, but no better performance was achieved. A smaller number of features suggested a simpler model. So *C* = 2.4 may be the best choice based on Figure 3. Its also interesting to observe that at least 155 features were chosen when *C* = 3.2, 3.4 and 3.5. So the following sections tried to find a smaller feature subset from this list of 147 features, which were listed in the Supplementary Table S1.

### Selecting Features by Filters

A filter algorithm assumed the inter-feature independence and evaluated each feature separately for its association with the phenotype. So the AFS strategy selected the *k*-feature subset as the top-ranked *k* features. While the DFS strategy removed the least-ranked feature from a (*k* + 1)-feature subset based on the filter-calculated single-feature association with the class label. That is to say, the *k*-feature subset generated by the DFS strategy was also the top-ranked *k* features. The ascending and DFS strategies of a filter algorithm selected the same features for a given number of features. So this section only investigated the AFS() strategy of the four filter algorithms. The details of the AFS strategy were described in the section “Heuristic Feature Selection Strategies.”

**FIGURE 4:**
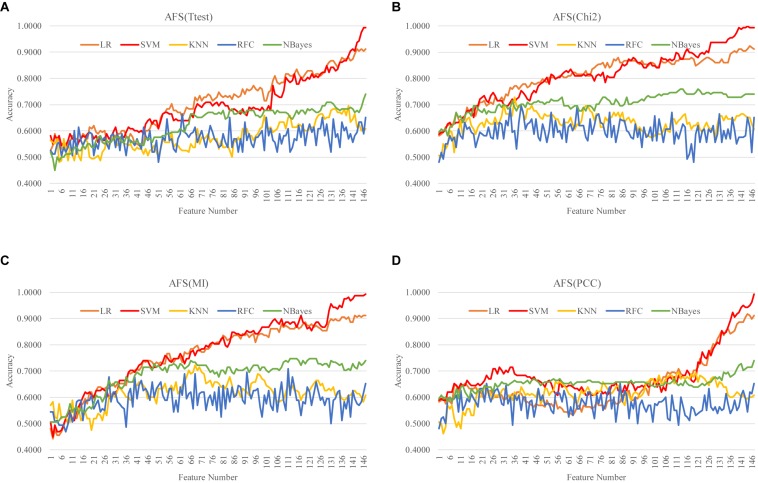
Ascending feature screening (AFS) of four filter algorithms. The classification performance of each filter algorithm was evaluated by five classifiers. The four filter algorithms were **(A)** Ttest, **(B)** Chi2, **(C)** MI, and **(D)** PCC, and the five classifiers were LR, SVM, KNN, RFC, and NBayes.

Our data suggested that all the five classifiers performed similarly well on a feature subset with a size <50, as shown in Figure 4. However, the two classifiers LR and SVM kept improving the classification accuracies by adding more features. And SVM achieved the best classification accuracies on features selected by all the four filter algorithms. The best model with Acc = 1.0000 was achieved by the classifier SVM with 144 Chi2-selected methylomic features. The other three classifiers (KNN, RFC and NBayes) reached the plateau of about 0.7000 in Acc after the number of features reached 50.

### Selecting Features by the RFE Strategies

We firstly evaluated the two feature selection procedures AFS(rfeLR) and DFS(rfeLR), as shown in Supplementary Figure S1. Filter algorithms had the assumption of the inter-feature independence. Although filters usually ran faster than the other algorithms like wrappers and RFE strategies, filters usually selected more features to achieve similar classification accuracies as the other feature selection algorithms (Srivastava et al., 2014; Suto et al., 2016).

When almost all the 147 features were kept, AFS(rfeLR) and DFS(rfeLR) performed similarly well for each of the five classifiers. The same pattern as in the previous section was observed that the two classifiers LR and SVM outperformed the other three with significantly improved accuracies, and the classifier SVM performed the best. Supplementary Figure S1 illustrated a novel pattern that the descendent feature removal strategy (DFS) performed much better than the ascendant feature addition strategy (AFS). AFS(rfeLR) required at least 116 features to achieve Acc > 0.9000. While DFS(rfeLR) only needed 41 features to achieve Acc = 0.9114.

**FIGURE 5:**
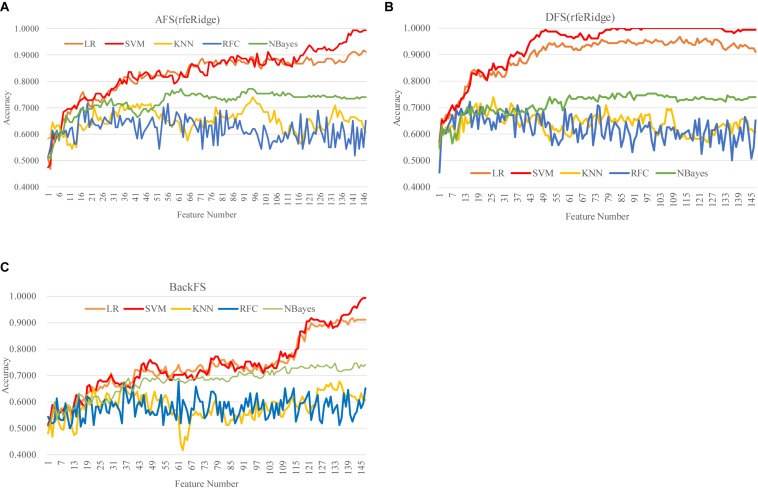
Comparison of the three feature screening strategies of the feature selection algorithm rfeRidge. The classification performance was evaluated by five classifiers. The five classifiers were LR, SVM, KNN, RFC, and NBayes. And the three feature screening strategies were **(A)** AFS(rfeRidge), **(B)** DFS(rfeRidge), and **(C)** BackFS.

DFS(rfeRidge) performed even better than AFS(rfeRidge), as shown in Figure 5 and Supplementary Figure S4. AFS(rfeRidge) selected 97 features to train an SVM model with Acc = 0.9051. But only 37 methylomic features were selected by DFS(rfeRidge) to train an SVM model with Acc = 0.9114. And the SVM model performed very stably with more features selected by DFS(rfeRidge), as shown in Figure 5. The strategy BackFS required many more features to achieve a similar prediction accuracy, as in Figure 5C. The classifier NBayes assumed the inter-feature independence, which may not be the case in the dataset used in this study. This might be the reason that the classifier NBayes didn’t perform very well in this study, as shown in Figure 5.

Also, DFS(rfeLasso) performed better than AFS(rfeLasso), as shown in Supplementary Figure S2. AFS(rfeLasso) selected 144 features to train an SVM model with Acc = 0.9684. But 144 methylomic features were selected by DFS(rfeLasso) to train an SVM model with Acc = 0.9810. And the SVM model performed very stably with more features selected by DFS(rfeLasso).

DFS(rfeNBayes) performed similarly well for each of the five classifiers as AFS(rfeNBayes), as shown in Supplementary Figure S3. Both AFS(rfeNBayes) and DFS(rfeNBayes) achieved Acc = 0.9177 when selecting 101 features to train an SVM model. And the SVM model performed very stably with more features selected.

Overall, the best model achieved in this study was the SVM model (Acc = 1.0000) using the 81 features selected by the strategy DFS(rfeRidge), as shown in Figure 5.

**FIGURE 6:**
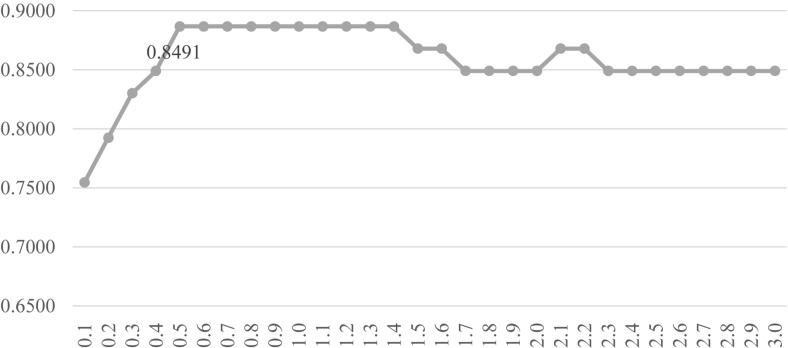
Parameter tuning for the SVM parameter *C*. The horizontal axis was the value of the parameter *C*, and the vertical axis was the classification accuracy. Each model was trained on the training dataset and evaluated on the validation dataset.

Another evaluation procedure was carried out for the above-selected features. The stratified splitting strategy was used to split the samples into one-third training, one-third validation, and one-third test datasets. The SVM parameter C was evaluated for its different values from 0.1 to 3.0 with the step size 0.1, as shown in Figure 6. After the 81 methylomic features were selected by the strategy DFS(rfeRidge), the binary classification SVM models with different *C* values were trained on the training dataset and evaluated for the classification accuracies on the validation dataset, as shown in Figure 6. When the parameter was 0.5, the validation accuracy reached the best value 0.8868. A similar classification accuracy 0.8679 was achieved on the test dataset. This suggested the model stability for the classification algorithm.

### Refining the 147 Features With Two Other Regression Algorithms

This study evaluated how the regression-based feature selection algorithms might be improved by two other regression algorithms, i.e., sliced inverse regression (SIR) (Cook and Weisberg, 1991; Li, 1991) and group lasso (GroupLasso) (Yuan and Lin, 2006; Yuan et al., 2011). Figure 1 demonstrated that the LinearSVC model selected 147 features and then the filters and regression-based RFE algorithms were applied. So SIR and GroupLasso were utilized to further refine the subset of 147 features.

Sliced inverse regression doesn’t need to optimize the parametric or non-parametric model training process and demonstrates a significant capability to reduce the feature dimensions (Cook and Weisberg, 1991; Li, 1991). This study utilized the SIR in the Python package sliced version 0.1 (Li, 1991). Its interesting to observe that the classifier SVM from the best model achieved again Acc = 1.0000 using only the first feature engineered by SIR. Our experimental data demonstrated that SIR and the proposed feature selection procedure achieved the same classification performances on the investigated problem in this study. But the best model used only 81 original methylated residues while SIR used the one feature engineered from the 147 features.

GroupLasso is another widely used feature selection algorithm that assigns non-zero weights to groups of features instead of the individual ones like the regular lasso (Yuan and Lin, 2006; Yuan et al., 2011). This study utilized GroupLasso in the Python package group-lasso version 1.1.1 (Yuan and Lin, 2006; Yuan et al., 2011). Unfortunately no features were selected by GroupLasso.

### Refining Differentially Methylated and Variable Biomarkers

Twenty differentially methylated residues were detected in the previous study, but all of them were not statistically significantly associated with RA by the adjusted *p*-values (Webster et al., 2018). This study further refined this subset of 20 methylation residues with the classification accuracy as the optimization goal.

The AFS strategy of the four filter algorithms was applied to the 20 differentially methylated residues, as shown in Supplementary Figure S5. The classifier NBayes achieved the best Acc = 0.7532 on the original subset of 20 features. This model may be further improved to Acc = 0.7658 using only 10 features, which was selected by the algorithm AFS(MI). Another algorithm AFS(Ttest) achieved the same prediction Acc = 0.7532 using only 4 and 10 features for the classifiers KNN and NBayes, respectively.

An even better improvement may be achieved by both AFS(rfeLasso) and DFS(rfeLasso), as shown in Supplementary Figure S6. Firstly, the original list of 20 differentially methylated residues may be reduced to 11 features to achieve Acc = 0.7658. Secondly, the best model achieved Acc = 0.8038 using only 18 features.

Webster et al. (2018) also evaluated a list of two differentially variable residues, which were refined in the same way in this study, as shown in Supplementary Figures S7, S8. The similar patterns were observed, and the best improved SVM model achieved Acc = 0.7722 with 12 features selected by AFS(Chi2).

### Refining the Previous Biomarkers by BackFS

**FIGURE 7:**
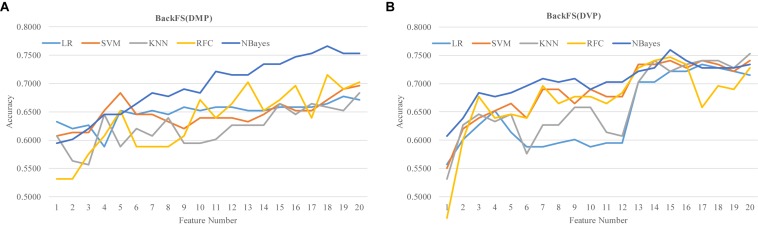
Refine the two lists of previous methylation biomarkers of RA. The classification performance was evaluated by five classifiers. The five classifiers were LR, SVM, KNN, RFC, and NBayes. Refining procedures of **(A)** the 20 differentially methylated positions (DMP) and **(B)** the 20 differentially variable positions (DVP).

The two lists of RA biomarkers were further refined by a simple iterative feature elimination procedure BackFS, as shown in Figure 7. BackFS exhaustively removed the redundant features, so only the subset of features achieving the best prediction accuracy was kept for further analysis. The original list of 20 differentially methylated features may be further selected to achieve a better prediction Acc = 0.7658 using only 18 features for the classifier NBayes, as shown in Figure 7A. While the list of 20 differentially variable features may be reduced to 15 with a better prediction Acc = 0.7595 for the same classifier NBayes, as shown in Figure 7B.

### Independent Effectiveness Evaluation of the Proposed Biomarker Detection Procedure

**FIGURE 8:**
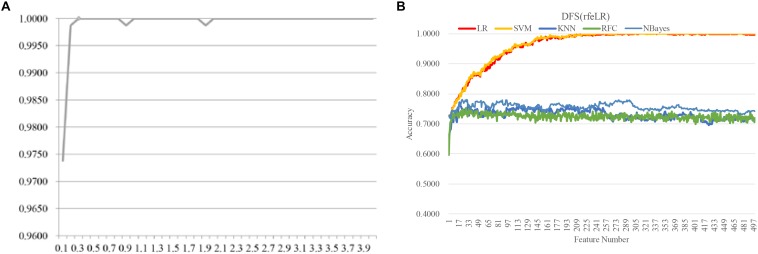
Evaluating the proposed biomarker detection procedure with a new dataset TCGA-BRCA. The classification performance was evaluated by five classifiers. The five classifiers were LR, SVM, KNN, RFC, and NBayes. **(A)** The classification accuracies (vertical axis) of the classifier LinearSVC with different values of the parameter *C* between [0.10, 4.00] with the step size 0.10. **(B)** The classification accuracy plots of the feature selection strategy DFS(rfeLR) using the five classifiers, i.e., LR, SVM, KNN, RFC, and NBayes.

We further evaluated the effectiveness of the proposed biomarker detection procedure on an independent dataset. There is no simulation tool for the array-based methylomes. So another independent dataset TCGA-BRCA (Berger et al., 2018) was chosen to evaluate our biomarker detection procedure, as shown in Figure 8. There were 982 samples and each sample had 485,577 methylated residues. Multiple samples were extracted from some patients and only sample was randomly chosen to represent this patient. 763 samples were collected to have the clinical annotation “tumor_stage” (I/II/III/IV). The binary classification problem was formulated between the class Positive (555 samples from the stages I and II) and Negative (208 samples from the stages III and IV).

The same biomarker detection procedure was carried out on the methylomic dataset TCGA-BRCA, as shown in Figure 6. The initial 20,000 top-ranked features with the largest standard-deviations were screened to find the best value of the parameter *C*, as shown in Figure 6. The binary classification problem for the dataset TCGA-BRCA seemed to reach the classification accuracy 1.0000 with the parameter *C* = 0.3. There were 499 features selected in this step. Then the four filter algorithms were evaluated using the AFS strategy and the four RFE algorithms were evaluated by both AFS and DFS strategies, in the same procedure as the above. The features screened by DFS(rfeLR) achieved the best classification accuracy 1.0000 using only 240 features. Among the five classifiers, SVM achieved the best performance, as the same in the RA biomarker detection problem. The best feature selection algorithm DFS(rfeRidge) for the RA biomarker detection problem achieved a similar classification accuracy (0.9882) for the dataset TCGA-BRCA.

So overall the biomarker detection procedure in this study effectively detected methylated residues for the methylome-based classification problems.

### Biological Observations of Methylomic Biomarkers

This study selected 81 methylated residues as biomarkers to separate the RA patients from their controls, as shown in Supplementary Table S1. Its interesting to observe that 38 of these 81 methylated residues were from the chromosome Y and many of them were within the transcriptional start sites (TSS) of non-coding RNA gene family Testis-Specific Transcript, Y-Linked (TTTY). This supported the observations in the literature about the gender discrepancy on autoimmune diseases like RA (Jansson and Holmdahl, 1994). Many of these methylated residues were in the TSS regions of these non-coding RNAs, suggesting that methylation may have played a regulatory role in the onset and development of RA (Relle et al., 2015; Houtman et al., 2018). Such reversible epigenetic modifications may serve as therapeutic candidates (Cribbs et al., 2015; Doody et al., 2017).

Another RA-associated gene HLA-DRB1 (Major Histocompatibility Complex, Class II, DR Beta 1) was also a methylation biomarker (cg27107292) detected in this study (Conigliaro et al., 2019; Okada et al., 2019). HLA-DRB1 was one of the first few RA biomarkers discovered four decades ago and harbored more than 100 RA-associated loci (Okada et al., 2019). Recently, HLA-DRB1 was also observed to be differentially methylated in RA (Liu et al., 2013) and had significant associations with the mortality and prognosis of RA (Ruyssen-Witrand et al., 2012; Viatte et al., 2015) and other autoimmune diseases (Bettencourt et al., 2012; Okayama et al., 2018). Furthermore, the pathway analysis through the KEGG Database (Kanehisa et al., 2017) demonstrated that various immune pathways were associated with HLA-DRB1 such as hsa04612 (Antigen processing and presentation pathway), hsa04659 (Th17 cell differentiation pathway), and hsa05323 (RA pathway). This suggested that the detected biomarker HLA-DRB1 was strongly connected to the autoimmune disease RA.

Furthermore, C5orf30 (a methylation biomarker cg17605604) was reported as a damaging regulator of tissue in RA, which is highly expressed in RA synovial fibroblast (RASF) involving joint destruction (Muthana et al., 2015). The clinical data analysis also demonstrated that the variant rs26232 in C5orf30 locus was testified to be associated with RA susceptibility and radiologic damage severity. These observations from the literature supported that C5orf30 may play a significant role in the progression of arthrosis damage (Teare et al., 2013).

Two gender-specific methylation biomarker genes DDX3Y and UTY which have been reported as sex-affected differentially expressed genes for inflammatory arthritis through the Wnt signaling (Kudryavtseva et al., 2012). This situation exactly matched to the gender-biased disease condition for RA. Besides DDX3Y was suggested to be differentially expressed in cartilage tissues of RA patients versus control groups with potential association with miRNA (Toraih et al., 2016). Many other genes like RPS4Y2, KDM5D, EIF1AY, and CYorf15A have also been shown as important biomarker genes in RA via the Monte Carlo cross-validation (Song et al., 2017).

Supplementary Table S1 also illustrated that the methylated biomarkers were from various genic sites, i.e., TSS, 5′-untranslated region (UTR), 3′-UTR, first exon, and genic body. This suggested that these RA methylation biomarkers contributed their regulatory roles through different biological mechanisms. Those frequently appeared genes, and non-coding RNA genes may need further wet-lab investigations of their potential biological mechanisms.

## Conclusion

This study comprehensively utilized the widely used modeling algorithms to find the set of methylomic features with the best RA prediction accuracy. The best model used the features selected by the DFS(rfeRidge) strategy and the classifier SVM. The best accuracy 100.00% was achieved with the 81 detected methylomic biomarkers using the 10FCV strategy. The 81 methylomic biomarkers may accurately separate the RA patients from their matched controls. These biomarkers also demonstrated that chromosome Y contributed 38 methylated residues to the final model, supporting the literature about the gender-specific discrepancy. These 81 methylated biomarkers came from both regulatory regions and the gene body. So the biological mechanisms of how these 81 methylated residues were involved in RA’s onset and development may vary from the transcriptional regulation to the epigenetic modifications.

The number of biomarker features was still too large for the clinical practice. Clinical data other than the methylomic features may be integrated to improve the proposed RA detection model. A weakened model may also be considered using fewer features. For example, if only 37 methylomic features selected by DFS(rfeRidge) were used to train the SVM model, the detection accuracy reached Acc = 0.9114, an acceptable accuracy in some cases. RA was a complex human disease and the subtypes may be described by fewer biomarkers. So the detection models for the RA subtypes may also use fewer biomarkers to achieve satisfying accuracies.

The samples were 70 pairs of monozygotic twins. Each twin shared the same genetic background that might reduce the noise information induced by the methylation status of genetic variations. This sample setting suggested that the detected methylomic biomarkers mainly reflected the epigenetic status of RA. Independent validation datasets might also further improve our models.

## Data Availability Statement

Publicly available datasets were analyzed in this study. This data can be found here: E-MTAB-6988 at the ArrayExpress database.

## Author Contributions

FZ and XF conceived the project and designed the experiments. XF, XH, RS, ZX, LH, and QY wrote the codes and conducted the experiments. XF, XH, RS, and ZX generated the experimental results and drafted the discussions. FZ and XF discussed the experimental design and polished the manuscript. FZ and XF drafted and polished the manuscript. FZ, QY, and XF designed and carried out the additional experiments according to the reviewers’ comments. FZ, QY, and XF also revised and polished the revised version of the manuscript.

## Conflict of Interest

The authors declare that the research was conducted in the absence of any commercial or financial relationships that could be construed as a potential conflict of interest.
