# Supplementary material for: Detection and Comparative Analysis of Methylomic Biomarkers of Rheumatoid Arthritis
Source: Front Genet. 2020 Mar 27;11:238. doi: 10.3389/fgene.2020.00238 (PMC7119472; doi:10.3389/fgene.2020.00238)
Supplement: Supplementary file 1 [file Data_Sheet_1.PDF]

Detection and comparative analysis of methylomic biomarkers of rheumatoid arthritis

Xin Feng[1,2,6], Xubing Hao[3], Ruoyao Shi[4], Zhiqiang Xia[2], Lan Huang[5], Qiong Yu[1,#], Fengfeng Zhou[2,#].

1 Department of Epidemiology and Biostatistics, School of Public Health, Jilin University, Changchun 130021, China

2 BioKnow Health Informatics Lab, College of Computer Science and Technology, and Key Laboratory of Symbolic Computation and Knowledge Engineering of Ministry of Education, Jilin University, Changchun, Jilin 130012, China.

3 BioKnow Health Informatics Lab, College of Software, and Key Laboratory of Symbolic Computation and Knowledge Engineering of Ministry of Education, Jilin University, Changchun, Jilin 130012, China.

4 BioKnow Health Informatics Lab, College of Life Sciences, Jilin University, Changchun, Jilin 130012, China.

5 College of Computer Science and Technology, and Key Laboratory of Symbolic Computation and Knowledge Engineering of Ministry of Education, Jilin University, Changchun, Jilin 130012, China.

6 Jilin Institute of Chemical Technology, Jilin, Jilin 132022, China.

# Correspondence may be addressed to Fengfeng Zhou: FengfengZhou@gmail.com or ffzhou@jlu.edu.cn . Lab web site: http://www.healthinformaticslab.org/ . Correspondence may also be added to Qiong Yu at yuqiong@jlu.edu.cn .

Supplementary Figure S1

Ascending and descending feature screening of the feature selection algorithm rfeLR. The classification performance was evaluated by five classifiers. The five classifiers were LR, SVM, KNN, RFC and NBayes. (a) AFS(rfeLR) and (b) DFS(rfeLR).

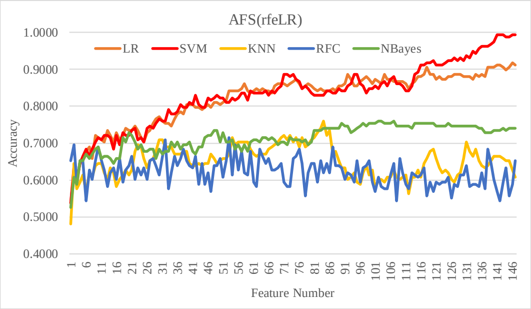

(a)

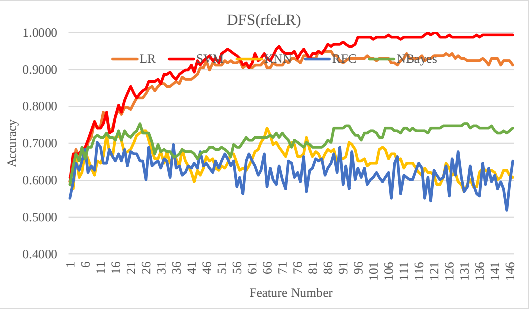

(b)

Supplementary Figure S2

Ascending and descending feature screening of the feature selection algorithm rfeLasso. The classification performance was evaluated by five classifiers. The five classifiers were LR, SVM, KNN, RFC and NBayes. (a) AFS(rfeLasso) and (b) DFS(rfeLasso).

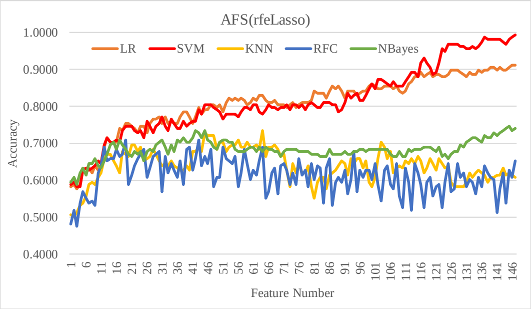

(a)

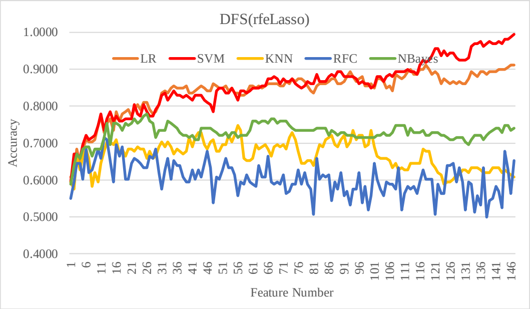

(b)

Supplementary Figure S3

Ascending and descending feature screening of the feature selection algorithm rfeNBayes. The classification performance was evaluated by five classifiers. The five classifiers were LR, SVM, KNN, RFC and NBayes. (a) AFS(rfeNBayes) and (b) DFS(rfeNBayes).

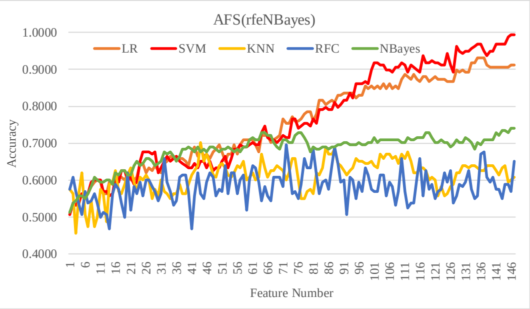

(a)

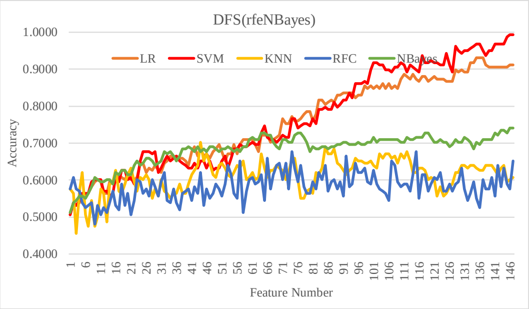

(b)

Supplementary Figure S4

Ascending and descending feature screening of the feature selection algorithm rfeRidge. The classification performance was evaluated by five classifiers. The five classifiers were LR, SVM, KNN, RFC and NBayes. (a) AFS(rfeRidge) and (b) DFS(rfeRidge).

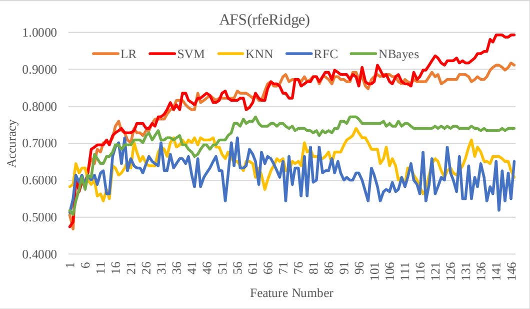

(a)

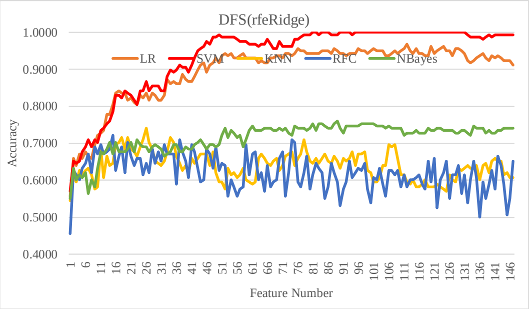

(b)

Supplementary Figure S5

Ascending feature screening of four filter algorithms on the two differentially methylated residues in the previous study. The classification performance of each filter algorithm was evaluated by five classifiers. The four filter algorithms were (a) Ttest, (b) Chi2, (c) MI, and (d) PCC, and the five classifiers were LR, SVM, KNN, RFC and NBayes.

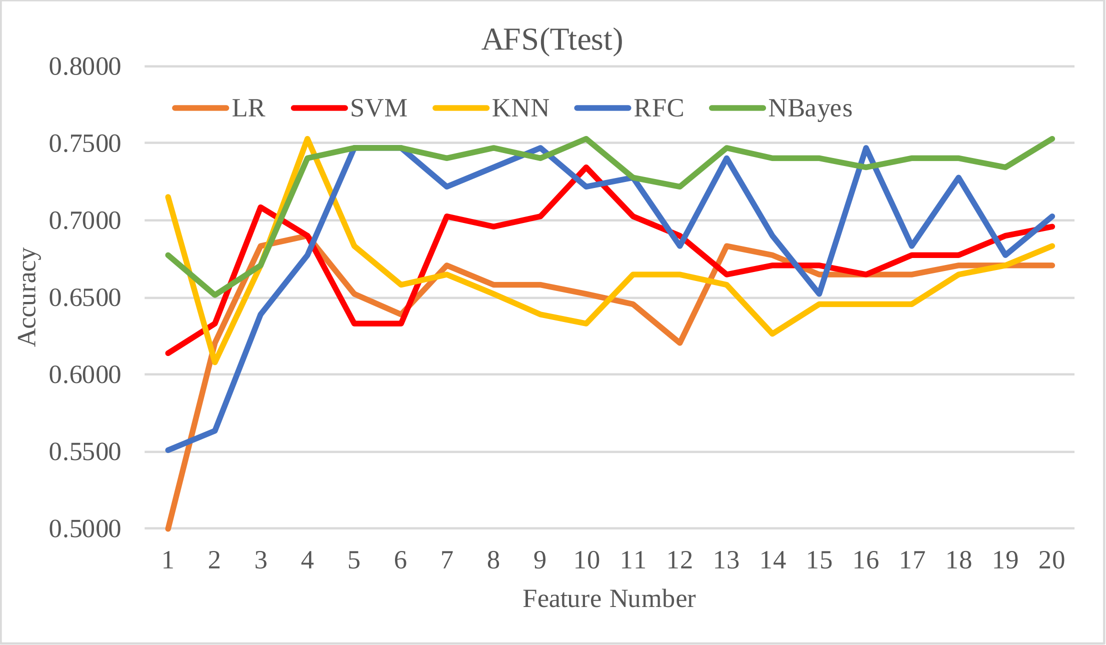

(a)

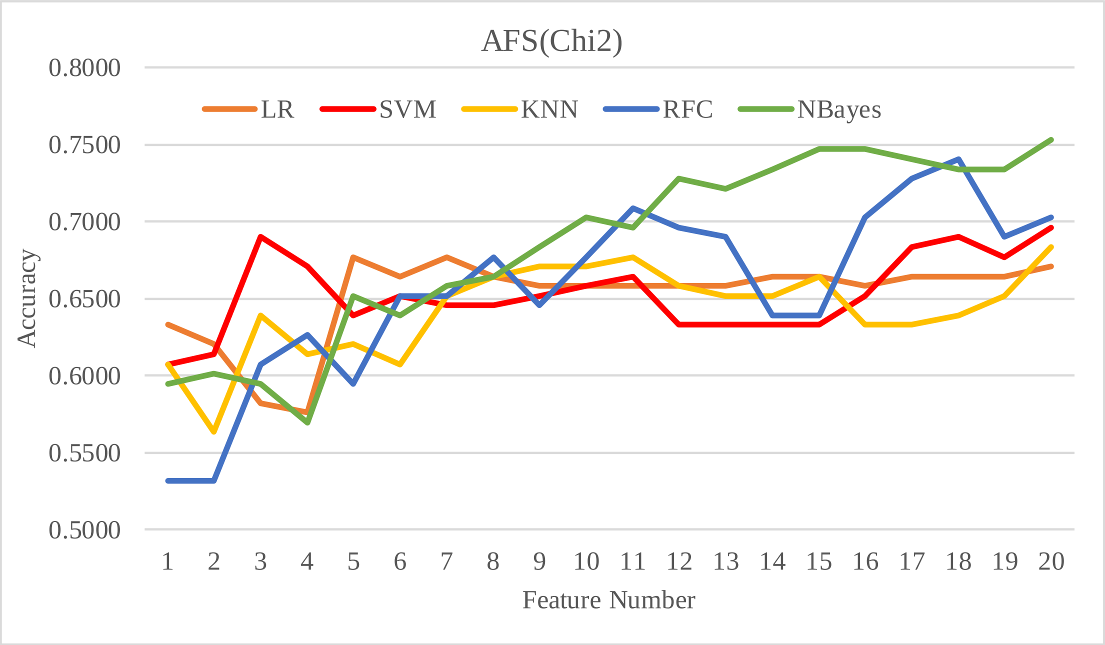
AFS(Chi2)
0.8000
0.7500
0.7000
0.6500
0.6000
0.5500
0.5000
LR
SVM
KNN
RFC
NBayes
Accuracy
1 2 3 4 5 6 7 8 9 10 11 12 13 14 15 16 17 18 19 20
Feature Number

(b)

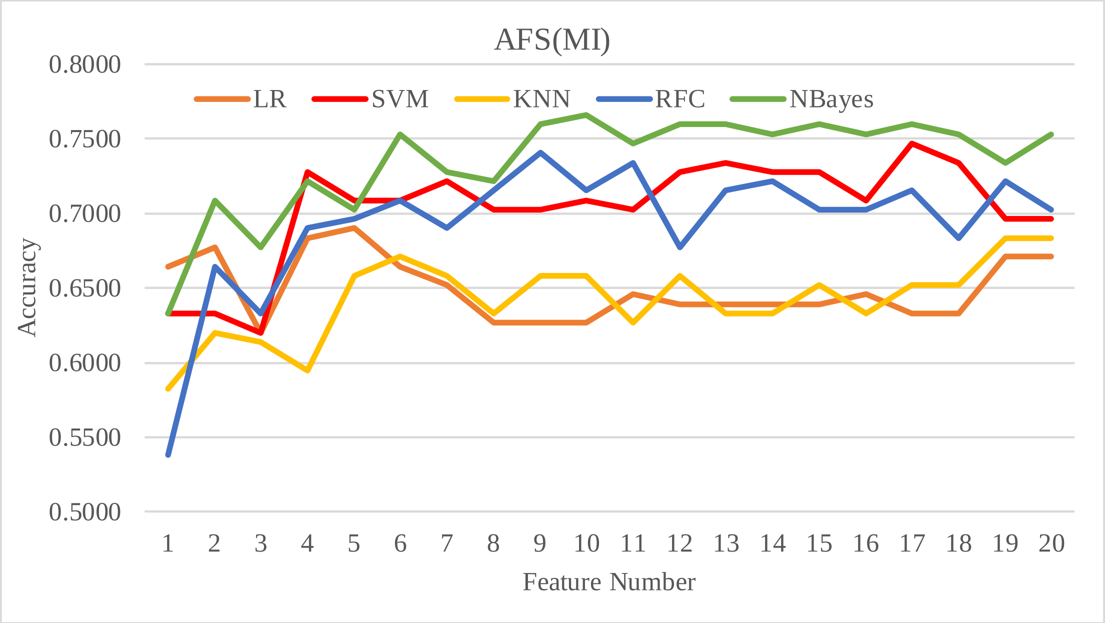
AFS(MI)
0.8000
0.7500
0.7000
0.6500
0.6000
0.5500
0.5000
LR
SVM
KNN
RFC
NBayes
Accuracy
1 2 3 4 5 6 7 8 9 10 11 12 13 14 15 16 17 18 19 20
Feature Number

(c)

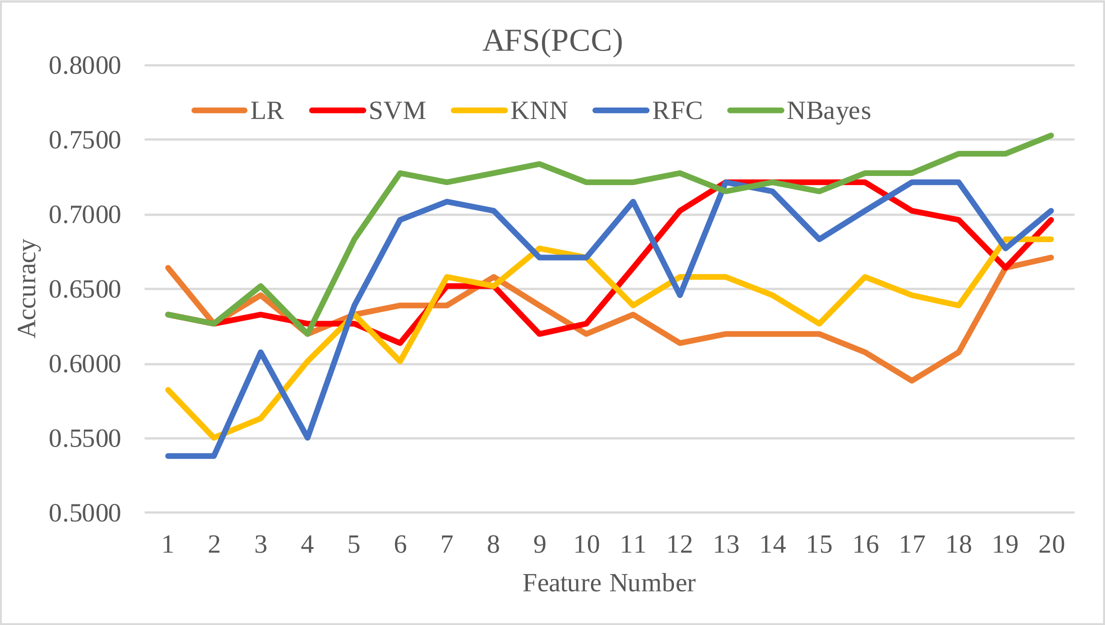

(d)

Supplementary Figure S6

Ascending and descending feature screening of the four RFE feature selection algorithms on the two differentially methylated residues in the previous study. The classification performance was evaluated by five classifiers. The five classifiers were LR, SVM, KNN, RFC and NBayes. (a) rfeLR, (b) rfeLasso, (c) rfeNBayes and (d) rfeRidge.

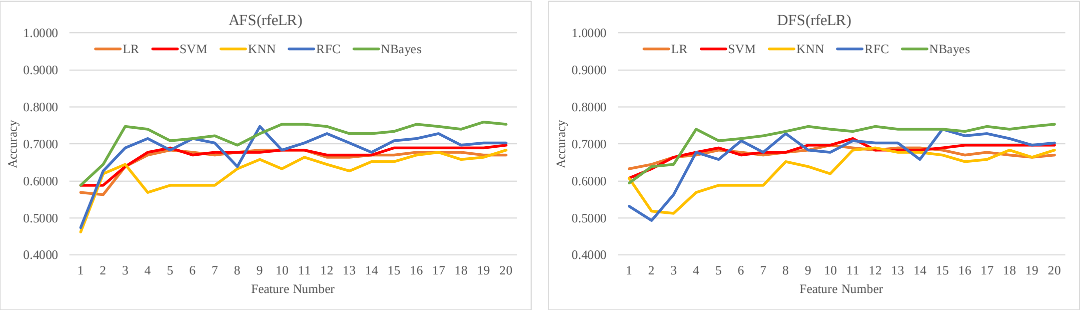

(a)

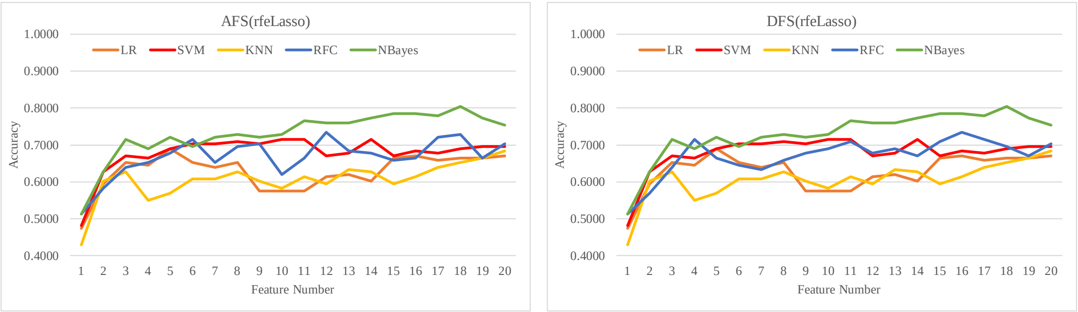

(b)

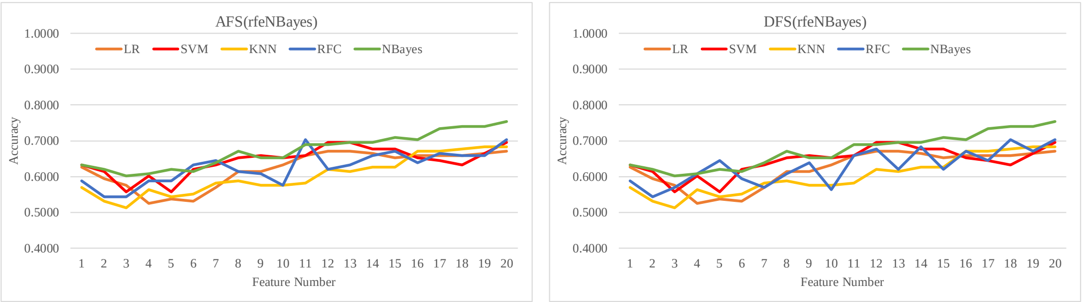

(c)

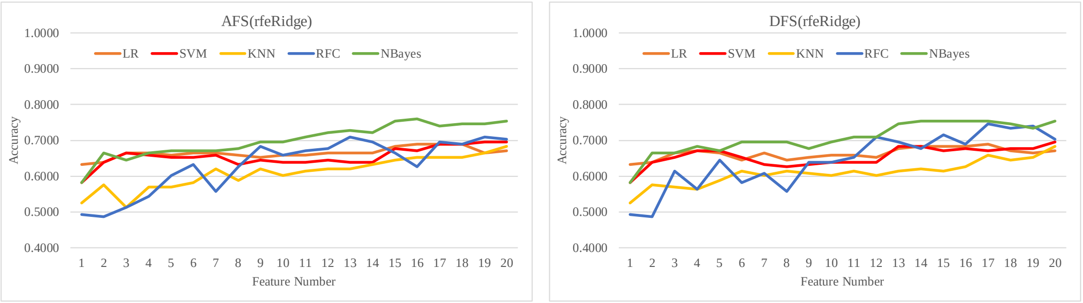

(d)

Supplementary Figure S7

Ascending feature screening of four filter algorithms on the two differentially variable residues in the previous study. The classification performance of each filter algorithm was evaluated by five classifiers. The four filter algorithms were (a) Ttest, (b) Chi2, (c) MI, and (d) PCC, and the five classifiers were LR, SVM, KNN, RFC and

NBayes.

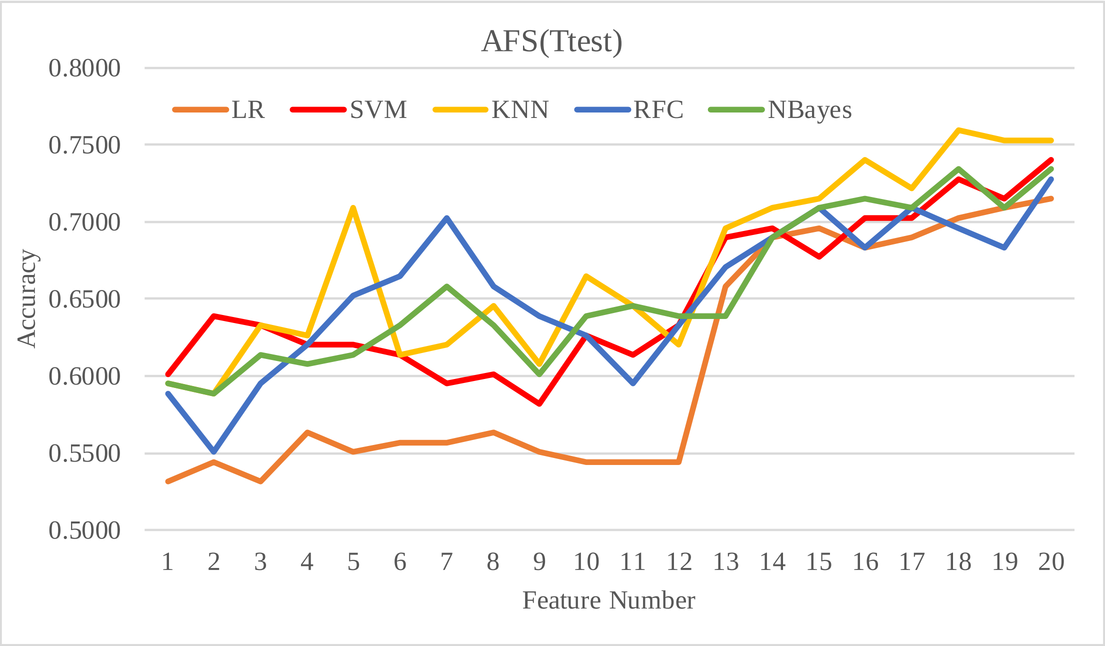

AFS(Ttest)
0.8000
0.7500
0.7000
0.6500
0.6000
0.5500
0.5000
LR
SVM
KNN
RFC
NBayes
Accuracy
1 2 3 4 5 6 7 8 9 10 11 12 13 14 15 16 17 18 19 20
Feature Number

(a)

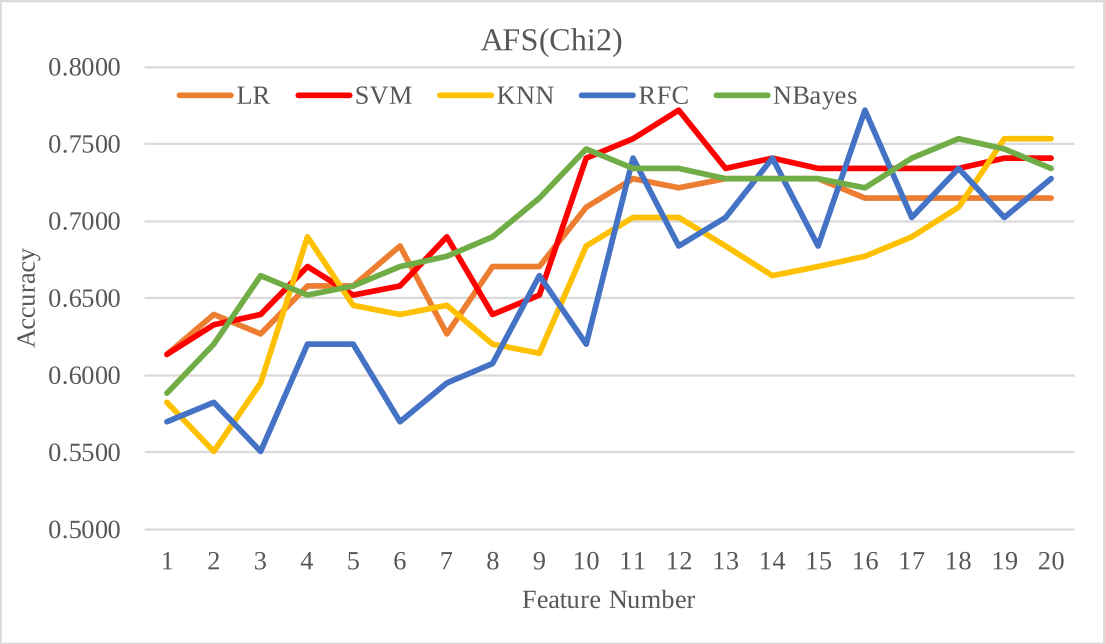

AFS(Chi2)
0.8000
0.7500
0.7000
0.6500
0.6000
0.5500
0.5000
LR
SVM
KNN
RFC
NBayes
Accuracy
1 2 3 4 5 6 7 8 9 10 11 12 13 14 15 16 17 18 19 20
Feature Number

(b)

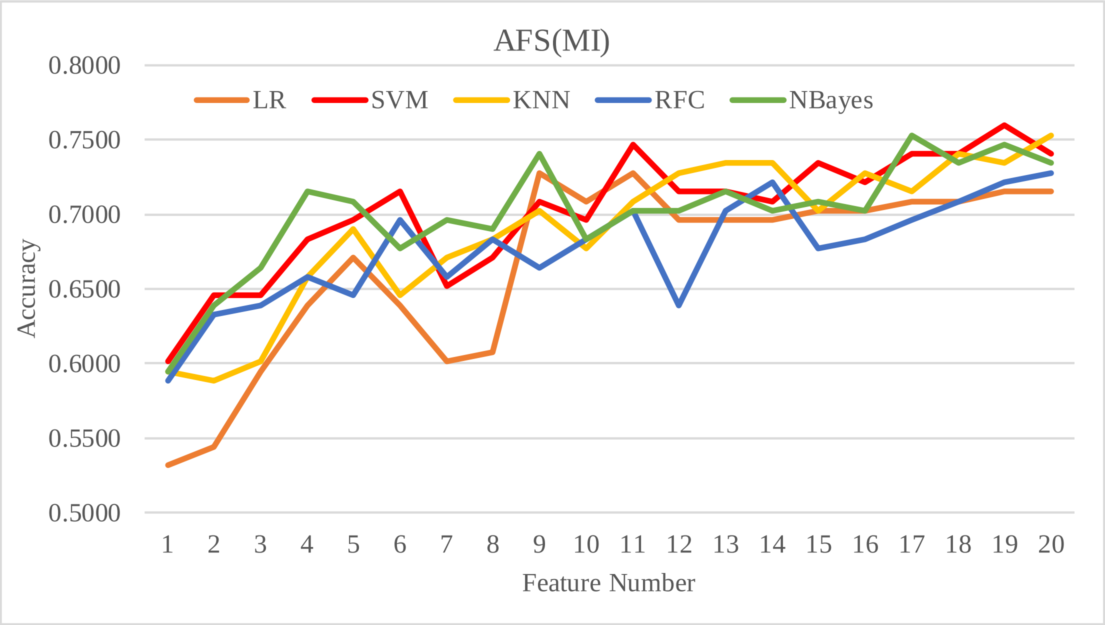
AFS(MI)
0.8000
0.7500
0.7000
0.6500
0.6000
0.5500
0.5000
LR
SVM
KNN
RFC
NBayes
Accuracy
1 2 3 4 5 6 7 8 9 10 11 12 13 14 15 16 17 18 19 20
Feature Number

(c)

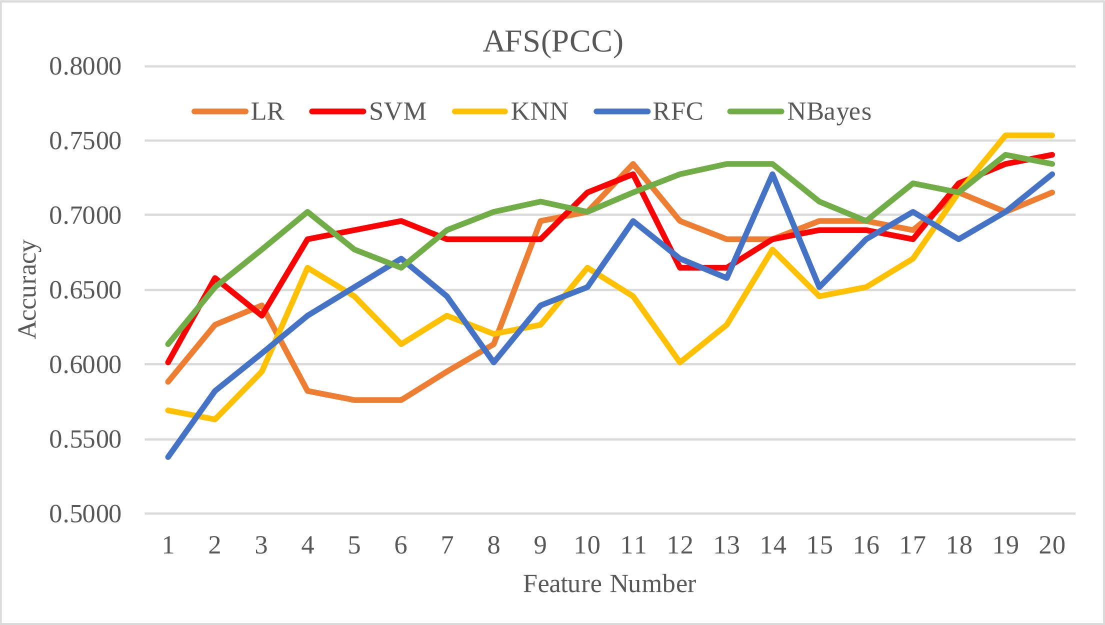
AFS(PCC)
0.8000
0.7500
0.7000
0.6500
0.6000
0.5500
0.5000
LR
SVM
KNN
RFC
NBayes
Accuracy
1 2 3 4 5 6 7 8 9 10 11 12 13 14 15 16 17 18 19 20
Feature Number

(d)

Supplementary Figure S8

Ascending and descending feature screening of the four RFE feature selection algorithms on the two differentially variable residues in the previous study. The classification performance was evaluated by five classifiers. The five classifiers were LR, SVM, KNN, RFC and NBayes. (a) rfeLR, (b) rfeLasso, (c) rfeNBayes and (d) rfeRidge.

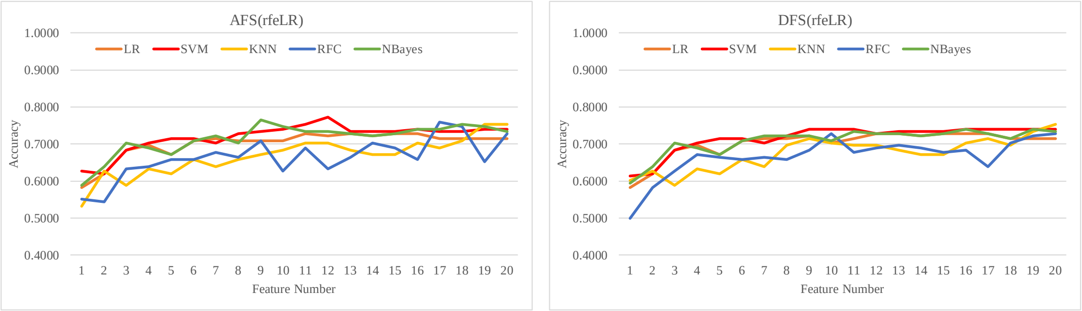

(a)

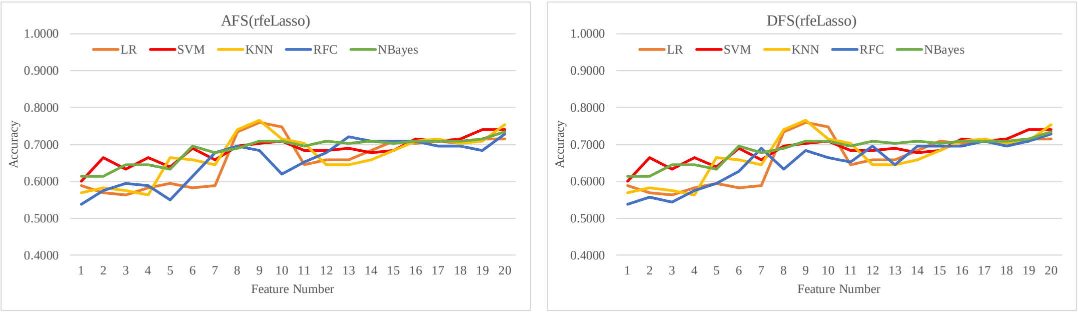

(b)

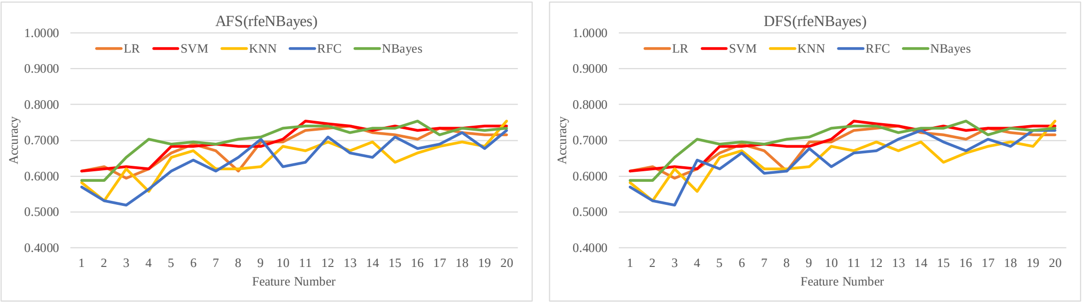

(c)

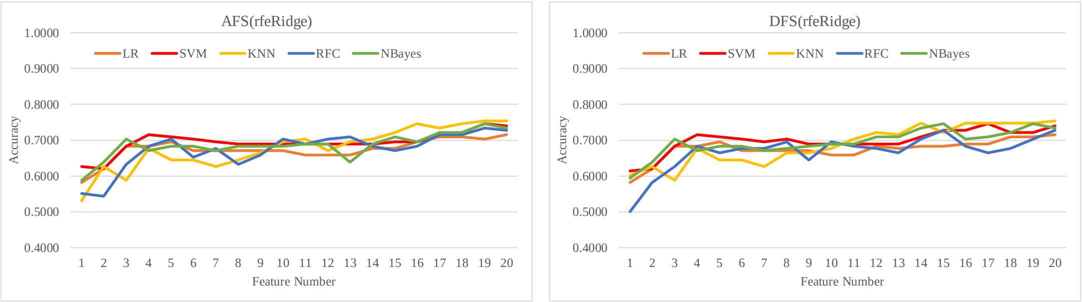

(d)

Supplementary Table S1

81 methylomic biomarkers for rheumatoid arthritis. The biomarker associated genes were also listed for the functional characterization of these methylomic biomarkers.

	Chr	Pos	Strand	RefGene	RefGene-Group	Enhancer
cg06388544	1	25598878	F	RHD;RHD	TSS200;TSS200	
cg05714732	1	32409096	F			
cg01419577	1	149170327	R			
cg13064658	1	212003989	R	LPGAT1;LPGAT1	5'UTR;1stExon	
cg02187090	2	47346813	F	C2orf61	Body	1
cg04277055	4	185749877	F			
cg18803147	5	2743124	R			
cg09819502	5	36242508	R	C5orf33;C5orf33	TSS1500;TSS1500	
cg17605604	5	102612899	F	C5orf30	3'UTR	
cg18409782	5	136243322	R			1
cg18236584	5	151476553	F			1
cg07225509	5	153622812	R	GALNT10	Body	1
cg11256764	5	180085446	R			
cg00955808	6	21754525	F	FLJ22536	Body	1
cg15415945	6	31627678	R	C6orf47	1stExon	
cg16564946	6	32304276	F	C6orf10	Body	
cg10995422	6	32522872	F	HLA-DRB6	Body	
cg27107292	6	32547167	R	HLA-DRB1	Body	
cg03649130	6	161456290	R	MAP3K4;MAP3K4	Body;Body	1

cg23727079	7	16966323	R			1
cg25909532	7	158821175	F	VIPR2	3'UTR	
cg02018764	8	2122276	F			
cg16866567	8	38759261	R	PLEKHA2	5'UTR	
cg07927909	8	39380592	R	ADAM3A;ADAM3A;ADAM3A	TSS200;TSS200;TSS200	
cg20426042	8	97778709	R	PGCP	5'UTR	1
cg12833683	8	100649114	F	VPS13B;VPS13B	Body;Body	1
cg18825597	8	144506958	R			
cg21211688	9	136403935	F	ADAMTSL2;ADAMTSL2	Body;Body	
cg17724121	11	5291531	F	HBE1	TSS200	
cg18518074	11	64642316	R	EHD1	Body	
cg21687840	11	128764920	R	KCNJ5	5'UTR	
cg19611616	12	27397833	R	STK38L	5'UTR	
cg07875068	12	116814302	R			1
cg02805689	13	111722259	R			
cg01317586	15	89921236	F	LOC254559	TSS200	
cg04246864	16	33357426	F			
cg00534215	16	89167792	R	ACSF3;ACSF3;ACSF3	Body;Body;Body	
cg00693004	17	43151433	F	NMT1	Body	1
cg14069205	17	67241141	R	ABCA10;ABCA5;ABCA5	TSS200;3'UTR;3'UTR	
cg16503797	18	19476805	R			
cg00843105	19	44645597	R	ZNF234;ZNF234	TSS200;TSS200	
cg11979743	20	814510	F	FAM110A;FAM110A	1stExon;5'UTR	
cg22666875	22	24348640	R	GSTTP1	TSS1500	
cg24837623	Y	2802900	R	ZFY;ZFY;ZFY	TSS1500;TSS1500;TSS1500	
cg04817258	Y	6117321	F			
cg04123665	Y	6131007	F			
cg13805219	Y	6137453	R			
cg00576139	Y	6312218	R	LOC100101115;TTTY21	Body;Body	
cg13419214	Y	6341961	R	TTTY8B;TTTY8	TSS1500;TSS1500	
cg20401549	Y	7141681	R	PRKY	TSS1500	
cg15781156	Y	7569967	F	TTTY16	TSS1500	
cg04831594	Y	8501021	R			
cg01828798	Y	8571457	R	TTTY19	TSS1500	
cg27325772	Y	9178472	F			
cg02606988	Y	9216456	F	TSPY4;TSPY4	Body;Body	
cg25705492	Y	9307448	F	TSPY4	Body	
cg14720093	Y	10033382	F			
cg02107461	Y	14103651	F			
cg18077436	Y	15016622	R	DDX3Y;DDX3Y	TSS200;5'UTR	
cg05940236	Y	15017297	R	DDX3Y;DDX3Y	Body;Body	
cg06479204	Y	15591533	R	UTY;UTY;UTY	1stExon;1stExon;1stExon	
cg02730008	Y	15814320	F	TMSB4Y	TSS1500	

cg26198148	Y	15814685	F	TMSB4Y	TSS1500	
cg08593141	Y	15866447	F			
cg04419680	Y	16637061	R	NLGN4Y;NLGN4Y;NLGN4Y	Body;5'UTR;Body	
cg16894943	Y	20488269	R	LOC401630;LOC401629	TSS200;TSS200	
cg15281205	Y	21100472	R	TTTY14	Body	
cg05230942	Y	21155270	R	CD24;TTTY14	TSS1500;Body	
cg10811597	Y	21239219	F	TTTY14	Body	
cg00212031	Y	21239348	R	TTTY14	TSS200	
cg15345074	Y	21239461	R	TTTY14	TSS200	
cg14492024	Y	21729049	R	CYorf15A	TSS200	
cg25815185	Y	21906868	F	KDM5D;KDM5D;KDM5D	TSS200;TSS200;TSS200	
cg10799208	Y	22681436	R	TTTY10	TSS1500	
cg10172760	Y	22736833	R	EIF1AY	TSS1500	
cg11225091	Y	22737663	R	EIF1AY;EIF1AY	5'UTR;1stExon	
cg03750315	Y	22737896	R	EIF1AY	Body	
cg01943289	Y	22917296	F	RPS4Y2	TSS1500	
cg06322277	Y	22917937	F	RPS4Y2	TSS200	
cg03416979	Y	23566288	R			
cg17939569	Y	27009430	F	DAZ2;DAZ4;DAZ3;DAZ2;DAZ4	Body;Body;Body;Body;Body	
